# Modulation of Neurotransmitter Pathways and Associated Metabolites by Systemic Silencing of Gut Genes in *C. elegans*

**DOI:** 10.3390/diagnostics13142322

**Published:** 2023-07-10

**Authors:** Shikha Shukla, Ankit Saxena, Sanjeev K. Shukla, Aamir Nazir

**Affiliations:** 1 Division of Toxicology and Experimental Medicine, CSIR-Central Drug Research Institute, Lucknow 226031, India; shukla04shikha@gmail.com; 2 Sophisticated Analytical Instrumentation Facility and Research Division, CSIR-Central Drug Research Institute, Lucknow 226031, India; ankit1507saxena@gmail.com; 3 Academy of Scientific and Innovative Research (AcSIR), Ghaziabad 201002, India

**Keywords:** *C. elegans*, HR-MAS, NMR, metabolites, neurotransmitter, gut genes, neuroprotection

## Abstract

The gut is now recognized as the “second brain” of the human body due to its integral role in neuronal health and functioning. Although we know that the gut communicates with the brain via immunological factors, microbial metabolites, and neurotransmitters, the interplay of these systems remains poorly understood. To investigate this interplay, we silenced 48 genes that are exclusively or primarily expressed in the *C. elegans* intestine. We studied the associated effects on various aspects of neurodegeneration, including proteotoxicity induced by α-Syn expression. We also assayed behaviours, such as mobility and cognition, that are governed by various neurotransmitters. We identified nine gut genes that significantly modulated these events. We further performed HR-MAS NMR-based metabolomics to recognize the metabolic variability induced by the respective RNAi conditions of *R07E3.1*, *C14A6.1*, *K09D9.2*, *ZK593.2*, *F41H10.8*, *M02D8.4*, *M88.1*, *C03G6.15* and *T01D3.6*. We found that key metabolites such as phenylalanine, tyrosine, inosine, and glutamine showed significant variation among the groups. Gut genes that demonstrated neuroprotective effects (*R07E3.1*, *C14A6.1*, *K09D9.2*, and *ZK593.2*) showed elevated levels of inosine, phenylalanine, and tyrosine; whereas, genes that aggravated neurotransmitter levels demonstrated decreased levels of the same metabolites. Our results shed light on the intricate roles of gut genes in the context of neurodegeneration and suggest a new perspective on the reciprocal interrelation of gut genes, neurotransmitters, and associated metabolites. Further studies are needed to decipher the intricate roles of these genes in context of neurodegeneration in greater detail.

## 1. Introduction

Neurodegenerative diseases are multi-factorial in nature and can be seen as an assemblage of various neurological disorders that may have pathological, clinical, or psychiatric manifestation [1]. In recent times, the developing comprehension of neurodegeneration and other biological pathways suggests that it is not entirely the brain that works as the central control for everything: even the brain has its retrograde signals from other systems to stay in check. One such demanding yet fully unexplored relation is that of how the gut and brain cross talk with each other [2]. At the anatomical level, the enteric nervous system forms the gut-brain axis [3], while the neuroendocrine system steers the signalling and maintenance of the axis. If each system is understood individually, then the nervous system participates in cognition to control and coordinate; however, when it faces any perturbation at genomic, transcriptomic, proteomic and homeostasis levels, diseases such as autism spectrum disorder, amyotrophic lateral sclerosis, transmissible spongiform encephalopathies, Parkinson disease (PD), and Alzheimer disease present as health wreckage. The gut plays crucial roles in nutrition assimilation and absorption and in signalling [4]. The gut also contains microbiota which contributes to endocrine signalling and immune response; likewise, the gut trains the host system and affects it via genetic and epigenetic modifications [5]. The Gut-Brain-Metabolism axis relies on metabolomic, metagenomic, transcriptomic, and epigenetic regulation. Each regulating factor has a vital role to play in its own domain. For example, metabolism and associated biochemical pathways can function as cues and hallmarks for an external stimulus-induced response, and sometimes it can be that the local host signalling can harmonize with the endocrine system [6]. This communication between gut and brain contributes to a new perspective on metabolism and associated disorders. If the signals come from the gut, what could be the possible triggering partners: maybe food, pH, endocrine signalling, etc. Thus, age links back to the nervous system, where it stands for instrumenting the whole metabolic response. The metagenomic domain harbours microbiota that in return supports the host, ranging from immunity to gene-expression regulation [7]. In recent studies, it is evident that there are few bacterial metabolites with recognised or speculated neuromodulator-activity determining signalling-pathway mechanisms from the gut microbiota to the brain that may have a prominent direct effect: e.g., gut bacterial cells possibly affecting specific brain cells [8,9,10,11]. Different gut metabolites having significant effects on neurovegetative pathology corroborates the theory of gut-brain connection: for instance, a study published recently demonstrates the role of the metabolite urolithin A in Alzheimer’s disease. This metabolome and gut microbiota are strongly interrelated in the neuronal set up at the individual level. On the other side, the transcriptome stays as a template for all gene expression and regulation which could be altered by a set of genes or an entirely different collection of genes with a differential organization in the host [12]. This network of differential expression connects every molecule in the organism to function in the dynamics of system homeostasis. The infamous molecular mimicry by gut microbiota and the subsequent modulation at the transcription level sets up a new level of understanding for the entire schema that runs with a centralized gut-brain axis [13]. There are several studies that demonstrate the roles of primary and secondary metabolites in neuronal health and their implications in neuronal ailments. Here, the enthralling question is: do gut and brain (e.g., the nervous system in *C. elegans*) coordinate with each other? If so, then by what means and how is it achieved? In the present study, we have investigated 48 genes that are abundantly or exclusively expressed in the intestines in context of various end points of ageing. We also conducted NMR-based metabolome studies to find the metabolites that are affected by these genes, to elucidate the putative reciprocity of gut genes, associated metabolites, and different end points of Parkinson’s Disease.

## 2. Materials and Methods

### 2.1. C. elegans Strains and Maintenance

Wild type (Bristol, UK) strain N2 and NL5901 [unc-54p::alphasynuclein::YFP + unc-119(+)] strain expressing “human” alpha-Synuclein protein in the muscles with YFP expression (obtained from the Caenorhabditis Genetic Center, University of Minnesota, St. Paul, MN, USA) were used in this study. *C. elegans* strains were cultured using standard protocols as described previously [14]. *Escherichia coli* (*E.coli*) strain OP50 was used as standard food to feed the worms. Synchronized worms (day 1) were obtained via an embryo-isolation procedure as described [14]. Briefly, worms were washed with M9 buffer and treated with axenizing solution (2 mL of sodium hypochlorite and 5 mL of 1 M sodium hydroxide solution) until eggs were released from the body.

### 2.2. dsRNA-Induced RNAi Silencing

The RNAi-induced systemic silencing of genes was performed as described here. Feeding protocol is one of the easiest ways to achieve the systemic silencing of respective genes. The bacterial clone having the dsRNA, intended to silence the respective genes (source: Ahringer library), was cultured for 6–8 h in LB and ampicillin (50 μg/mL). Once there was enough growth of the bacterial clone, it was seeded onto NGM plates. NGM plates had 5 mM IPTG and 25 mg/L carbenicillin. The plates were kept at 37 °C for 6–8 h in order to form bacterial lawns.

### 2.3. Thrashing Assay

This assay is performed in order to visualize the mobility of the worms in various treatment conditions. To perform this, firstly, worms were washed two to three times using M9 buffer. We placed individual worms on a glass slide containing 10 µL of M9 buffer and allowed them to rest for 30 s before commencing the count of their thrashes. The number of thrashes was counted for 30 s in every treatment group. The thrashing assay is one of the most effective behavioural assays; it indirectly indicates the levels of dopamine, and its concentration in *C. elegans* [15], as thrashes are regulated by a dopamine-signalling pathway.

### 2.4. Imaging of Human α-Synuclein Protein Expression

To visualise the effects exerted by the systemic silencing of various genes on α- Synuclein expression, worms were cultured onto IPTG plates having colonies of specific bacterial clones expressing dsRNA targeted to silence respective genes. In order to perform imaging, worms were washed with M9 buffer three times to get rid of all the bacteria. Worms were collected in a tube and centrifuged. We treated the worms with 100 mM of sodium azide (Sigma, Cat. No. 71289, Darmstadt, Germany) to immobilise them. After the immobilisation of the worms, the imaging was carried out employing a fluorescence microscope (Carl Zeiss Axio Imager M3, Jena, Germany). The YFP tagged α-Synuclein expression was quantified using ImageJ software, and from every treatment group, ten worms were arbitrarily selected for visualisation and to quantify the fluorescence intensity.

### 2.5. Aversion Assay

In *C. elegans*, various behavioural responses are regulated by a dopamine-signalling pathway. The aversion response to odorant nonanol is also tuned by dopamine signalling. When a nonanol drop is placed in close proximity to the head of a worm, the worm senses it and averts back as a chemotactic “aversive” response. In order to determine the systemic silencing effect of intestinal genes on the DA-related functions, we took advantage of an odor-based repellent assay using 1-nonanol, as described in a previous study [16]. Using this assay, we studied the response times of control- and treated-worms to 1-nonanol. After treatment of 48 h, worms were washed with M9 buffer thrice. In the beginning, worms were transferred to a sterile NGM agar plate without bacteria and allowed to crawl freely, to remove bacteria from the worm. A drop of 1-nonanol was placed near the head of a worm and the response times of the worms to the repellent were measured. At least 20 worms for each assay were counted. For all measurements, at least two biological replicates were performed, with data shown as mean  ±  SEM, and the statistical significance was evaluated using the Student’s *t* test.

### 2.6. NMR Sample Preparation

Samples of *C. elegans* worms viz. *F41H10.8*, *M02D8.4*, *M88.1*, *C03G6.15*, *T01D3.6*, *R07E3.1*, *C14A6.1*, *K09D9.2*, and *ZK593.2* were taken to observe their metabolic variation induced by the RNAi’s systemic silencing of screened genes, employing HR-MAS NMR spectroscopy. For sample preparation, worms were washed with M9 buffer, followed by fixation with paraformaldehyde solution, then washed three times with distilled water, and finally washed two times with D_2_O. Samples (population 5000) were stored at −80 °C, and then, while the NMR studies were performed, thawed at room temperature and placed in a 4 mm zirconia rotor with 50 µL capacity, followed by adding 20 µL D_2_O containing 0.355 mM TSP (3-trimethylsilylpropionic acid), that serves to lock the spectrometer and TSP as a reference [17].

### 2.7. NMR Experimental Condition

NMR experiments were performed using a Bruker Avance II 400 MHz NMR spectrometer operating at a proton NMR frequency of 400.13 MHz, equipped with a 5 mm HR-MAS ^13^C^−1^H Z gradient probe with a magic-angle gradient and sample-spinning rate of 4000 ± 1 Hz at 298 K. Since biological samples are mainly water, the NOESYPR1D NMR process was followed for better water suppression. The Carr-Purcell-Meiboom-Gill (CPMGPR1D) [RD-90°-{τ-180°-τ}n acquisition] pulse sequence was performed to reduce the intensity of macromolecules and lipids and to enhance the identification of small molecular-weight metabolites. All 1D 1H data were acquired using the following settings: spectral sweep width 20.54 ppm, relaxation delay 3 s, data points 32 K, flip angle of radiofrequency pulse 90°, and 256 scans. Pulse width P1 (15 µs) and pulse power Pl9 (56.76 dB) were optimised by using command pulsecal. Manual-phase correction and baseline correction were performed using Bruker Topspin 2.1 software, and chemical shifts were referenced concerning the TSP signal at δ 0.00 ppm. The residual water signal in the region (4.75 to 4.90 ppm) was excluded in the Chenomx (NMR suite 8.6 professional) processor module [18,19].

## 3. Results

### 3.1. Screening of 48 Gut Genes on the Basis of Behavioural Assays That are Regulated by Dopamine and ACh

Distinct motor functions are associated with the neuronal mechanisms and levels of neurotransmitters in *C. elegans* [20]. Neurotransmitters and their levels at neuromuscular junctions precisely regulate various behavioural aspects in the worms; dopamine regulates number of thrashes and chemorepellent behaviour in *C. elegans* [16]. To know the putative gut-gene targets, we performed aversion and thrashing assays that estimate the concentration of dopamine in neurons and at neuromuscular junctions. These assays indicated the target genes that alter motor behaviour of the worms, allowing us to further study them in terms of metabolome and reveal the interplay of dopamine concentration in neurons with metabolome in the gut. The genes that showed significant alterations in neuropathology are listed in (Table 1).

#### 3.1.1. Aversion Assay

**Figure 1:**
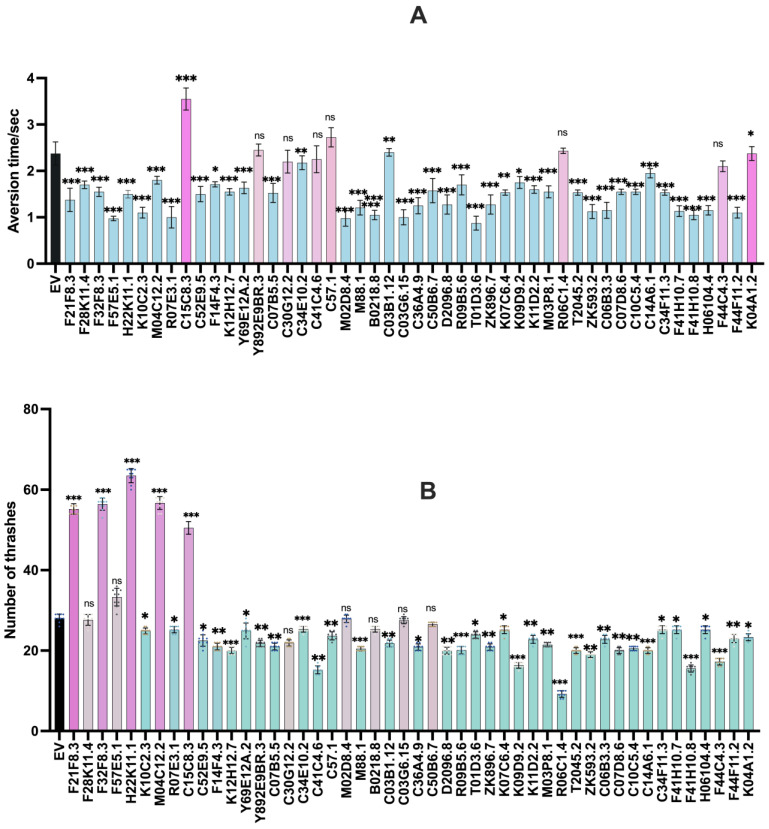
Knockdown effects of intestinal genes and their impact upon motility and aversion: (**A**) dsRNA-induced RNAi systemic silencing of 48 gut genes and its effect upon time taken to respond to nonanol exposure in transgenic strain NL5901. Significance was determined using Student’s *t*-test * *p* < 0.05, ** *p* < 0.01, *** *p* < 0.001 and ns—non-significant. (**B**) Effect of RNAi systemic silencing of the 48 genes on the number of thrashes in transgenic strain NL5901 of *C. elegans*. Significance was determined using Student’s *t*-test * *p* < 0.05, ** *p* < 0.01, *** *p* < 0.001 and ns—non-significant. (Black bar refers to control, Upregulated groups are colored pink, downregulated groups are colored green and non-significant groups are colored light brown).

Parkinson’s disease is characterised by compromised cognition and by impaired movements resulting from imbalance of various neurotransmitters including dopamine [21]. In *C. elegans,* various behavioural responses are regulated by the dopamine-signalling pathway. The aversion response to odorant nonanol is also tuned by dopamine signalling. When nonanol is kept in close proximity to the head of a worm, the worm senses it and averts back as a chemotactic “aversive” response. When there is any alteration in the dopamine concentration and thereafter in the dopamine-signalling pathway, the time taken to respond to the stimulant is altered [22]. We employed the aversion assay to screen genes that modulate the aversion response time following exposure to nonanol. We found that there were nine genes that showed alterations in the response time, as evident from Figure 1A. *R07E3.1*, *C14A6.1*, *K09D9.2*, *ZK593.2*, and *T01D3.6* showed significantly increased response times; whereas, *F41H10.8*, *M02D8.4*, *M88.1*, and *C03G6.15* demonstrated quicker responses to nonanol exposure (Figure 1A).

#### 3.1.2. Thrashing Assay

Neurotransmitter imbalances are one of the factors that are responsible for impaired movement in Parkinson’s disease patients [23]. In *C. elegans,* mobility is controlled by a fine balance between the excitatory and inhibitory neuronal signalling regulated by dopamine. Hence, we employed a thrashing assay to quantify the motility of worms. Here, the numbers of thrashes were counted for a specified amount of time; a single thrash was defined as the bending of the body to the outermost angle and then back to the initial posture. We found that there were nine genes that affected the number of trashes induced by RNAi treatment. *F41H10.8*, *M02D8.4*, *M88.1*, and *C03G6.15* caused significantly increased number of thrashes; whereas, *R07E3.1*, *C14A6.1*, *K09D9.2*, *ZK593.2*, *and T01D3.6* caused significantly fewer thrashes (Figure 1B).

### 3.2. dsRNA-Induced RNAi Systemic Silencing of Gut Genes in Transgenic Model of C. elegans Altered α-Synuclein Expression

**Figure 2:**
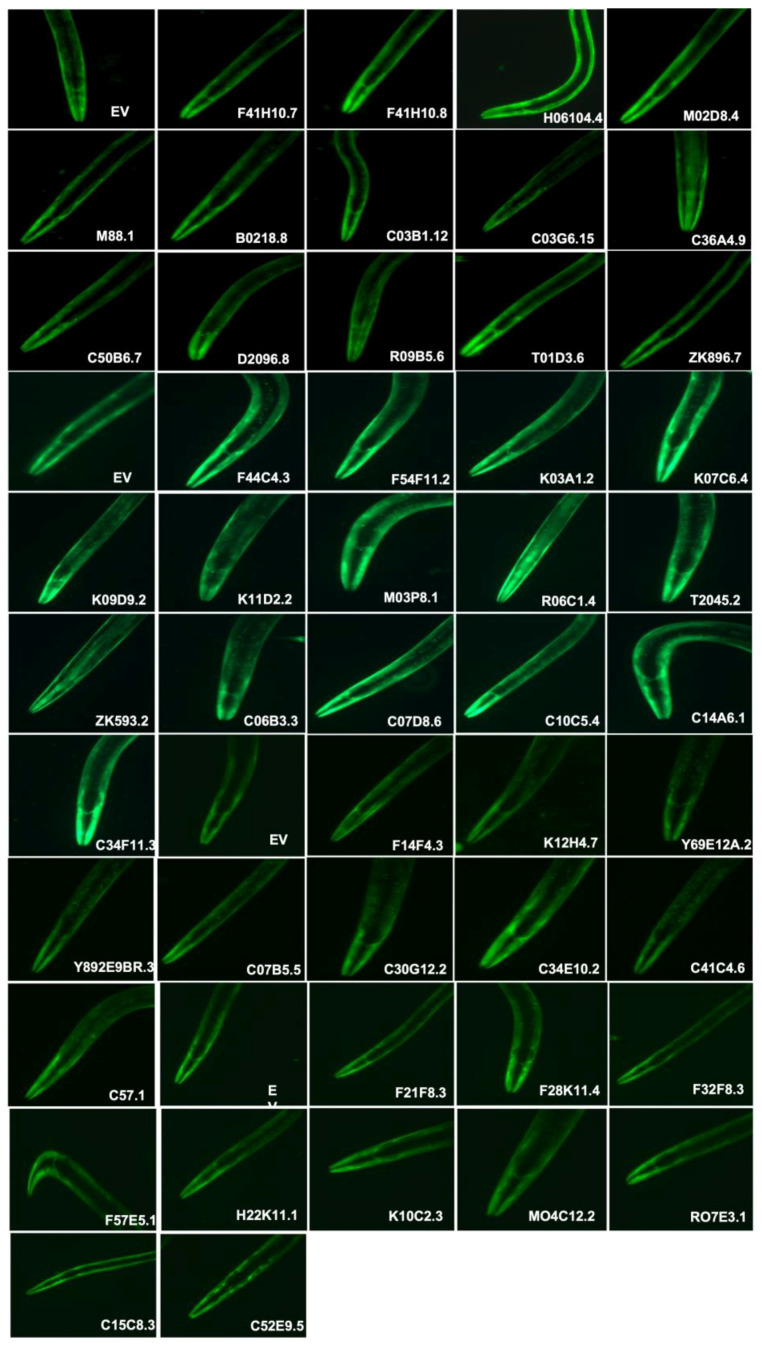
Systemic silencing of 48 gut genes and their effects on α-Synuclein expression: The expression of α-Synuclein was assayed in NL5901 [unc54p:: α-Synuclein:: YFP + unc-119(+)], this transgenic strain expresses human α-Synuclein intact with its muscles. All images used the same exposure time and gain settings.

It has been demonstrated in a study that α-Synuclein aggregation starts in the gut and is later transported into the brain via the vagus nerve connecting the gut with the brain [24,25]. Lately, gut-microbiota and their impacts on various biological processes have become quite intriguing all over the world as various studies have corroborated the important contribution of microbiota in reference to various disease conditions [26]. We performed a systematic RNAi screening of genes that are primarily expressed in the gut and studied these in context of the dopamine concentrations and various metabolites that participate in the formation of neurotransmitters including dopamine [27]. We performed dsRNA-induced RNAi systemic silencing of 48 gut genes and observed their effect upon α-Synuclein aggregation in transgenic strain NL5901. Out of 48 studied genes, the RNAi of nine genes (*R07E3.1*, *C14A6.1*, *K09D9.2*, *ZK593.2*, *T01D3.6*, *F41H10.8*, *M02D8.4*, *M88.1*, *C03G6.15*) showed significant alteration in α-Synuclein aggregation (Figure 2). As demonstrated in Figure 2, wild type EV showed an expression of 13.97 ± 0.2372 (*N* = 20). RNAi of *R07E3.1*, *C14A6.1*, *K09D9.2*, and *ZK593.2* showed 1.7, 1.9, 1.3, 1.6, and 2.0-fold upregulation; whereas, RNAi of *F41H10.8*, *M02D8.4*, *M88.1*, and *C03G6.15* showed 2.2, 1.7, 4.0, 1.4-fold downregulation of the protein expression.

### 3.3. Characterization of Metabolites

**Figure 3:**
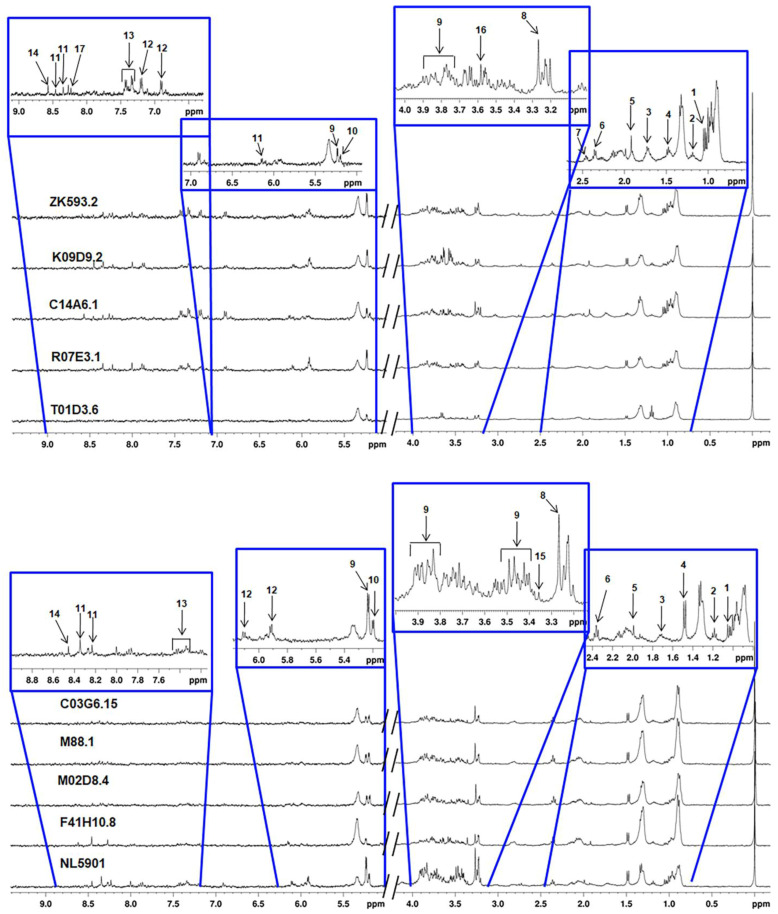
CPMG stacked plot of different *C. elegans* worm sample with assigned metabolites.

Assigned NMR spectra of *C. elegans* worm samples are shown in Figure 3. Metabolites were unambiguously identified and quantified using the Chenomx profiler module, which include alanine, acetate, betaine, glucose, glutamine, glutamate, glycine, inosine, lactate, leucine, lysine, methanol, phenylalanine, trehalose, tyrosine, uridine and valine. Further chemical shift values, coupling constants, and splitting patterns of these metabolites were compared with reported literature values [28], as well as with various databases i.e., Biological Magnetic Resonance Bank (BMRB, http://www.bmrb.wisc.edu accessed on 25 November 2020) and Human Metabolome Database (HMDB, http://www.hmdb.ca accessed on 25 November 2020) [29]. Concentration of these metabolites was expressed in terms of mean value ± standard deviation (SD) (Table 2 and Table 3).

### 3.4. Statistical Analysis

**Figure 4:**
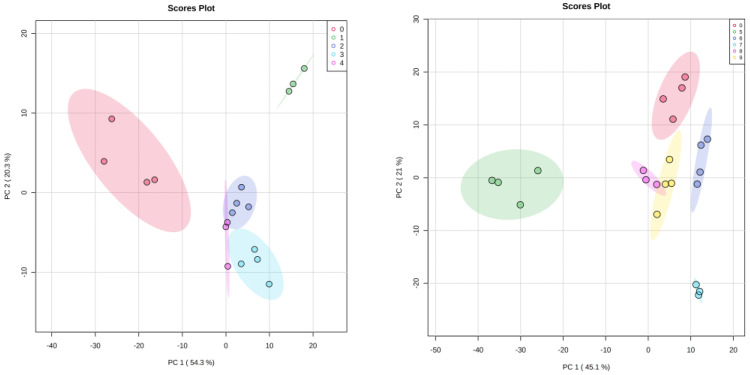
2D PCA score plot of *C. elegans* where Group–0 to Group–4 corresponds to NL5901, *F41H10.8*, *M02D8.4*, *M88.1*, and *C03G6.15* respectively, and Group–5 to Group–9 corresponds to *T01D3.6*, *R07E3.1*, *C14A6.1*, *K09D9.2*, and *ZK593.2* respectively.

**Figure 5:**
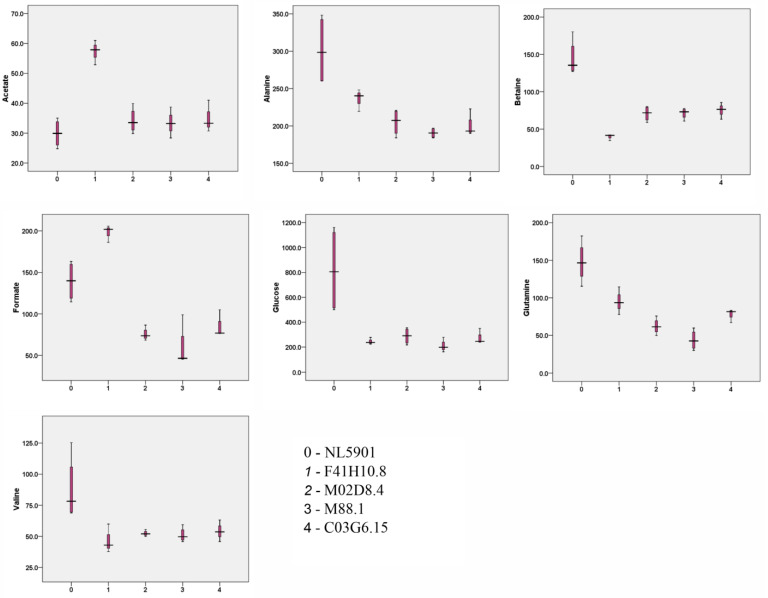
Box-whiskers graphical representation of significantly different metabolites (*p* < 0.05) of *C. elegans* worms.

**Figure 6:**
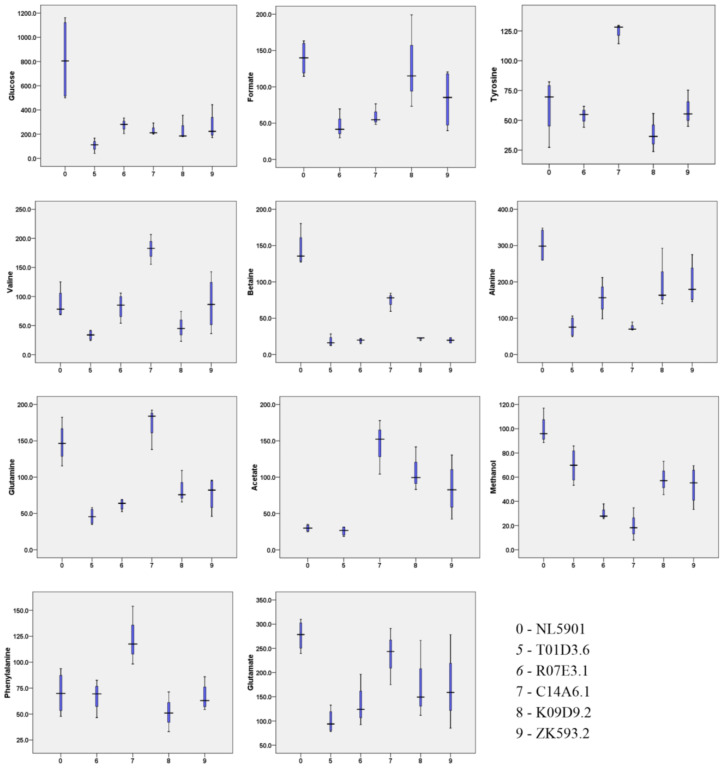
Box-whiskers graphical representation of significantly different metabolites (*p* < 0.05) of *C. elegans* worms.

Statistical analysis of concentration-profiled data (obtained from Chenomx) was carried out using Metaboanalyst 4.0 (www.metaboanalyst.ca accessed on 21 January 2021), an online web-based server [18]. Based on the effects of dsRNA-induced RNAi systemic silencing of gut genes upon various molecular events of neurodegeneration, we identified two classes of genes, one corresponding to genes that demonstrated positive regulation of neurotransmitters and a second corresponding to genes that demonstrated negative regulation of neurotransmitters. The first class of these genes includes NL5901, *F41H10.8*, *M02D8.4*, *M88.1* and *C03G6.15*, while the second class of genes includes NL5901, *T01D3.6*, *R07E3.1*, *C14A6.1*, *K09D9.2*, and *ZK593.2*. An unsupervised principal-component analysis was performed to identify the independent source of variance in terms of principal components (PC), and the obtained 2D score plot showed a separation trend among the groups. In the first batch, PC1 and PC2 in the 2D score plot explain 74.6% of the total variance, where PC1 contributed 54.3% and PC2 contributed 20.3% of the explained variance. In the second batch, the total variance in terms of PC1 and PC2 was 66.1%, where PC1 contributed 45.1% and PC2 contributed 21% of explained variance (Figure 4). For multiple-group analysis, one-way analysis of variance (ANOVA) was done using SPSS 16.0 software to determine the overall statistical significance of metabolites. Metabolites having statistical significance of *p* < 0.05 are represented in a box-whiskers plot (Figure 5 and Figure 6) [18].

### 3.5. HR-MAS NMR Spectroscopy Based Metabolome Profiling Strongly Reflects the Reciprocity of Gut Homeostasis and Neuronal Health

The high-resolution magic-angle spinning (HR-MAS) NMR spectroscopic technique has its own utility for direct analysis of various biological samples (cells, tissues, etc.), including minimal sample preparation for metabolomics. In the present analysis, it was observed that some metabolites including phenylalanine, tyrosine, inosine, glutamine, glutamate, etc., show significant variation among the groups. These metabolites play an important role in neurodegenerative diseases [30]. As reported earlier, glutamate is a key metabolite used by nerve cells to communicate information, but excessive glutamate signalling leads to excitotoxicity, that causes various neurodegenerative diseases, including Parkinson’s disease. The present study revealed the up-regulation of glutamate in groups *F41H10.8*, *M02D8.4*, *M88.1*, and *C03G6.15* and down-regulation in *T01D3.6*, *R07E3.1*, *C14A6.1*, *K09D9.2*, and *ZK593.2.* At the same time, glutamine specifically increases in RNAi of *C14A6.1*. Inosine, a naturally occurring purine nucleoside that acts as an antioxidant and possesses neuroprotective effects [31], was found in RNAi of *R07E3.1*, *C14A6.1*, *K09D9.2*, and *ZK593.2*, but diminished in RNAi of *F41H10.8*, *M02D8.4*, *M88.1*, *C03G6.15*, and *T01D3.6* compared to the NL5901 strain. It was also observed in the present study that phenylalanine and tyrosine [32] (precursors of monoamine neurotransmitters) were present in RNAi of *R07E3.1*, *C14A6.1*, *K09D9.2*, and *ZK593.2*, yet these metabolites were negligible after knockdown of *F41H10.8*, *M02D8.4*, *M88.1*, *C03G6.15*, and *T01D3.6*. Metabolic variation revealed up-regulated α-synuclein expression in groups *F41H10.8*, *M02D8.4*, *M88.1*, *C03G6.15*, and *T01D3.6*, while down-regulated α-synuclein expression in groups *R07E3.1*, *C14A6.1*, *K09D9.2*, and *ZK593.2* were observed (Table 2 and Table 3).

## 4. Discussion

The gut is now being regarded as the “second brain” of the body. Several studies and accumulating evidence provide strong insights into the correlation of metabolites, gut homeostasis, and neuronal health. The present study aimed to investigate the potential relationship among 48 gut genes and their impact on neurotransmitter metabolites. We found evidence of a reciprocal relationship between the systemic silencing of gut genes and alterations in neurotransmitter metabolites, indicating a possible regulatory role of gut genes in neurotransmitter levels. There are several plasma metabolites which are associated with biomarker evidence of a decline in cognition and neurodegeneration [33]. Metabolites belonging to biogenic amines (creatinine, asymmetric dimethylarginine; ADMA, kynurenine, trans-4-hydroxyproline), amino acids (proline, arginine, asparagine, phenylalanine, threonine, citrulline), and acylcarnitine classes have shown a synergistic relation with plasma neurofilament light chains. Additionally, metabolites of the kynurenine pathway have also shown positive correlations in context of blood markers of neurodegeneration [34]. In recent years, a unique perspective of pathology has emerged that lays claim to the pivotal role of gut homeostasis in neuronal health and consequently neurodegenerative diseases. Gut metabolites are produced during bacterial metabolic processes and can either be intermediates or end products [35]. Aromatic amino acids and short-chain fatty acids (SCFAs) are among the essential metabolites that are produced through the bacterial breakdown of dietary sources such as fruits and fibres, as well as endogenous metabolites derived from cholesterol and bile acids [36]. The primary means of signalling for these metabolites is through the autonomic nervous system (ANS) via the vagal and spinal nerves [37]. Additionally, these metabolites can enter the bloodstream and pass through the blood-brain barrier (BBB) to affect regulatory mechanisms in the central nervous system (CNS). Gut metabolites can also alter the gene expression of gut genes, and can thus regulate several pathways critical for diseases, including energy metabolism, the immune system, and others [35]. Earlier, we had demonstrated that an antiporter that is expressed exclusively in intestine of *C. elegans* elicits neuroprotective effects and ameliorates the toxic effects exerted by α-Synuclein aggregation, via mimicking calories restriction. Our findings prompted us to study other gut proteins that are involved in the maintenance of homeostasis of the gut in context of neurodegeneration. There are 48 such genes, which are primarily/exclusively expressed in the intestines of adult worms, and their homologues are present in humans. Considering the roles of alpha-synucleine aggregation and dopamine decline in PD [38], we studied these 48 genes in reference to alteration in transgenic expression of human α-Synuclein and behavioural assays that are governed by the dopamine-signalling pathway, to perform a screening of those genes that affect these parameters. Different studies have demonstrated that α-Synuclein aggregation might change the metabolite profiling in the gut [39]. A study conducted on monkeys showed significantly elevated levels of L-aspartic acid, p-hydroxyphenylacetic acid, and glyceric acid, hence our idea here was to see whether alteration in levels of these genes can affect the parameters of neurodegeneration or neuronal health. Out of the nine screened genes, four genes (*F41H10.8*, *M02D8.4*, *M88.1*, and *C03G6.15*) were ameliorating the dopamine levels as well as α-Synuclein expression; whereas, five genes (*T01D3.6*, *R07E3.1*, *C14A6.1*, *K09D9.2*, and *ZK593.2*) aggravated the toxicity induced by α- Synuclein expression and dopamine concentration. *F41H10.8* is predicted to enable fatty acid elongase activity, *M02D8.4* is predicted to activate the asparagine synthase activity, *M88.1* is predicted to enable glucuronosyltransferase activity; whereas, *C03G6.15* is involved in oxidoreductase activity, *T01D3.6* is predicted to enable calcium-ion binding activity, *R07E3.1* is predicted to enable cysteine type endopeptidase activity, *C14A6.1* is predicted to enable signalling-receptor activity, *K09D9.2* is predicted to have heme-binding activity, and *ZK593.2* is affected by several genes involved in calorie restriction such as *DAF-2*, *Eat-2* and others (https://wormbase.org/#012-34-5 accessed on 20 August 2020). Most of these genes are involved in metabolic processes and participate in maintaining gut homeostasis via regulating the metabolome of the gut. Food ingestion and modulation of nutrients function as cues to modulate the proteins that are primarily expressed in the gut, and these genes in turn change the metabolome which affects neuronal health and neurotransmission [24]. Hence, in the present study we employed HR-MAS based NMR spectroscopy to understand the metabolic variations that contribute to formation of various neurotransmitters in *C. elegans*. This metabolic profiling strongly shows that the genes that aggravated the toxicity of α-Synuclein aggregation showed decreased concentration of metabolites that participate in the formation of various transmitters. This result indicates that genes in the gut can alter the metabolites of several pathways including those that are involved in the formation of various neurotransmitters. When the precursors of these neurotransmitters are altered, that in turn reflects the alteration of neurotransmitter levels themselves. We found a reciprocity between the levels of neuroprotective metabolites and dopamine levels in worms. This is a unique idea that demonstrates a co-relationship between levels of metabolites and their possible implications in context of Parkinson’s disease. Our results presented herein unfold an intriguing finding, that neuroprotective effects exerted by these gut genes show reciprocity with the levels of metabolites, and offers a new perspective of the gut-metabolite-neuronal axis.

## Figures and Tables

**Table 1:** List of genes that show significant alterations in neuropathology.

*F41H10.8*	elo(Predicted to enable fatty acid elongase activity)
*M02D8.4*	asns(Predicted to enable asparagine synthase (glutamine-hydrolyzing) activity)
*M88.1*	ugt(Predicted to enable glucuronosyltransferase activity)
*C03G6.15*	cyp-35A2(Predicted to enable heme binding activity; oxidoreductase activity)
*T01D3.6*	Predicted to enable calcium ion binding activity
*R07E3.1*	Predicted to enable cysteine-type endopeptidase activity
*C14A6.1*	clec-48(Predicted to enable signaling receptor activity.
*K09D9.2*	cyp-35A3(Predicted to enable heme binding activity; oxidoreductase activity)
*ZK593.2*	Is affected by several genes including *daf-2*; *daf-12*; *eat-2*

**Table 2:** Concentration (µM) of the metabolites observed in *C. elegans* worms.

S. No.	Metabolites	Concentration (µM)				
		NL5901 (Mean ± SD)	*F41H10.8* (Mean ± SD)	*M02D8.4* (Mean ± SD)	*M88.1* (Mean ± SD)	*C03G6.15* (Mean ± SD)
1	Valine	87.6 ± 26.3	46.9 ± 11.6	52.4 ± 2.4	51.2 ± 5.9	54.2 ± 8.7
2	Ethanol	136.5 ± 33.7	-	-	-	104.2 ± 14.3
3	Leucine	139.5 ± 75.8	83.0 ± 43.6	66.5 ± 22.4	71.9 ± 17.5	85.5 ± 15.2
4	Alanine	301.4 ± 47.6	236.0 ± 14.8	204.9 ± 17.6	190.6 ± 6.6	201.9 ± 18.2
5	Acetate	29.9 ± 4.7	57.2 ± 4.1	34.2 ± 4.3	33.4 ± 4.2	35.0 ± 5.4
6	Glutamate	276.5 ± 32.0	399.5 ± 25.3	322.9 ± 62.1	327.9 ± 38.3	369.1 ± 62.4
7	Glutamine	147.8 ± 27.6	95.4 ± 18.4	62.1 ± 10.7	43.8 ± 13.3	77.3 ± 9.0
8	Betaine	144.7 ± 24.6	39.6 ± 4.1	70.8 ± 10.1	71.2 ± 7.4	75.1 ± 11.3
9	Glucose	818.2 ± 351.8	246.2 ± 28.1	288.5 ± 65.8	209.4 ± 49.5	278.6 ± 61.8
10	Trehalose	133.2 ± 16.1	-	74.9 ± 9.7	65.7 ± 14.7	55.8 ± 22.0
11	Inosine	85.3 ± 20.7	-	-	-	-
12	Tyrosine	62.2 ± 24.6	-	-	-	-
13	Phenylalanine	70.4 ± 20.7	-	-	-	-
14	Formate	139.4 ± 24.0	198.0 ± 10.2	75.6 ± 7.7	59.3 ± 26.3	86.1 ± 16.3
15	Methanol	99.4 ± 12.4	194.0 ± 11.3	96.5 ± 17.0	96.9 ± 7.1	64.4 ± 20.6
16	Glycine	68.2 ± 28.8	-	-	69.2 ± 14.4	67.2 ± 14.7

**Table 3:** Concentration (µM) of the metabolites observed in *C. elegans* worms.

S.No.	Metabolites	Concentration (µM)				
		*T01D3.6* (Mean ± SD)	*R07E3.1* (Mean ± SD)	*C14A6.1* (Mean ± SD)	*K09D9.2* (Mean ± SD)	*ZK593.2* (Mean ± SD)
1	Valine	33.6 ± 8.8	82.7 ± 22.5	181.7 ± 25.7	47.5 ± 25.7	88.0 ± 46.3
2	Ethanol	468.2 ± 145.8	-	-	163.3 ± 48.3	109.2 ± 47.5
3	Leucine	51.3 ± 9.3	82.8 ± 18.7	187.6 ± 36.2	46.0 ± 14.4	101.9 ± 20.5
4	Alanine	76.4 ± 28.3	155.7 ± 46.6	75.8 ± 12.0	198.7 ± 82.3	194.9 ± 58.7
5	Acetate	25.8 ± 6.4	-	144.8 ± 37.4	108.1 ± 30.2	84.6 ± 36.7
6	Glutamate	99.6 ± 25.2	134.2 ± 44.2	236.5 ± 58.4	175.9 ± 80.6	170.5 ± 79.9
7	Glutamine	46.0 ± 11.4	62.5 ± 7.9	171.5 ± 29.2	83.7 ± 22.8	76.5 ± 23.4
8	Betaine	18.2 ± 7.2	19.2 ± 3.2	74.0 ± 12.9	21.8 ± 2.1	19.6 ± 4.0
9	Glucose	107.7 ± 52.0	274.7 ± 53.2	234.8 ± 50.4	240.3 ± 100.1	265.3 ± 121.4
10	Trehalose	-	-	40.0 ± 17.0	-	-
11	Inosine	-	61.2 ± 18.6	42.5 ± 3.5	46.1 ± 26.4	33.3 ± 13.8
12	Tyrosine	-	53.9 ± 7.3	124.0 ± 8.5	38.7 ± 16.1	57.8 ± 12.78
13	Phenylalanine	-	67.0 ± 15.0	123.3 ± 28.3	51.8 ± 19.1	66.6 ± 13.8
14	Formate	-	45.6 ± 16.9	59.9 ± 14.8	129.2 ± 64.2	82.7 ± 41.3
15	Methanol	69.7 ± 14.7	29.8 ± 5.5	20.3 ± 13.4	58.6 ± 13.9	53.4 ± 15.9
16	Glycine	39.4 ± 13.0	84.0 ± 18.9	126.8 ± 47.0	150.0 ± 144.6	99.5 ± 25.1
17	Uridine	-	76.7 ± 36.8	-	59.5 ± 35.1	46.2 ± 9.5

## Data Availability

The data that support the findings of this study are available from the corresponding author upon reasonable request.
